# Glial pannexin1 contributes to tactile hypersensitivity in a mouse model of orofacial pain

**DOI:** 10.1038/srep38266

**Published:** 2016-12-02

**Authors:** Regina Hanstein, Menachem Hanani, Eliana Scemes, David C. Spray

**Affiliations:** 1 Dominick P. Purpura Department of Neuroscience, Albert Einstein College of Medicine, Bronx, NY, 10461, USA; 2 Laboratory of Experimental Surgery, Department of Surgery, Hadassah University Hospital, Jerusalem, Israel

## Abstract

Drug studies in animal models have implicated pannexin1 (Panx1) in various types of pain, including trigeminal hypersensitivity, neuropathic pain and migraine. However, the tested drugs have limited specificity and efficacy so that direct evidence for Panx1 contribution to pain has been lacking. We here show that tactile hypersensitivity is markedly attenuated by deletion of Panx1 in a mouse model of chronic orofacial pain; in this model, trigeminal ganglion Panx1 expression and function are markedly enhanced. Targeted deletion of Panx1 in GFAP-positive glia or in neurons revealed distinct effects. Panx1 deletion in GFAP-positive glia cells prevented hypersensitivity completely, whereas deletion of neuronal Panx1 reduced baseline sensitivity and the duration of hypersensitivity. In trigeminal ganglia with genetically encoded Ca^2+^ indicator in GFAP-positive glia or in neurons, both cell populations were found to be hyperactive and hyper-responsive to ATP. These novel findings reveal unique roles for GFAP-positive glial and neuronal Panx1 and describe new chronic pain targets for cell-type specific intervention in this often intractable disease.

Pathological pain is characterized by amplified response to noxious stimuli (hyperalgesia) and/or to normally innocuous stimuli (allodynia). The sequence of events leading to chronic pain include initial injury processes that are thought to generate sensitization and hyper-excitability of primary afferent neurons (termed peripheral sensitization), which in turn send nociceptive signals to the CNS producing central hypersensitivity that can long outlast the initial insult^1^,^2^,^3^,^4^. Chronic pain is generally managed with opiates, which are poorly effective and exhibit debilitating side-effects^5^,^6^. Thus, identifying molecular mechanisms underlying the development of chronic pain is essential to achieve the discovery of novel therapeutic targets.

Experimental data suggest that Pannexin 1 (Panx1) may be a new target for the development of non-narcotic medication to treat chronic pain. In the nervous system, Panx1 channels are expressed in neurons and glia^7^,^8^,^9^,^10^; upon activation, Panx1 channels release the algogenic molecule ATP and functionally and structurally associate with components of the inflammasome leading to cytokine production^9^,^11^,^12^. Evidence from animal models indicates that Panx1 plays a pivotal role in several types of pain, including trigeminal hypersensitivity^4^,^13^, spared nerve injury- and chemotherapy-induced neuropathic pain^14^,^15^,^16^, and migraine^17^. Recent studies performed on rodents treated with Panx1 inhibitors have indicated that these channels are likely involved in peripheral and central sensitization^14^,^16^. However, no Panx1 inhibitor is absolutely specific for Panx1, allowing the possibility that other molecular components may be affected by these drugs^18^. Thus the role of Panx1 in pain mechanisms is still obscure, and to address this issue, we undertook studies to evaluate the role of Panx1 in chronic pain using transgenic mice. We here describe experiments in which global and targeted cell-type specific deletion of Panx1 were compared with wildtype (WT) mice in our previously characterized orofacial pain model^4^, which is based on the injection of Complete Freund’s Adjuvant (CFA) into the submandibular skin (SMS) that results in activation of trigeminal ganglia. The outcome of this study, the first to examine pain sensitivity in Panx1 null mice, provides the first direct evidence that Panx1 contributes to orofacial pain and that glial and neuronal Panx1 differentially impact the threshold and persistence of tactile hypersensitivity.

## Results

### Deletion or blockade of Panx1 prevents allodynia

**Figure 1:**
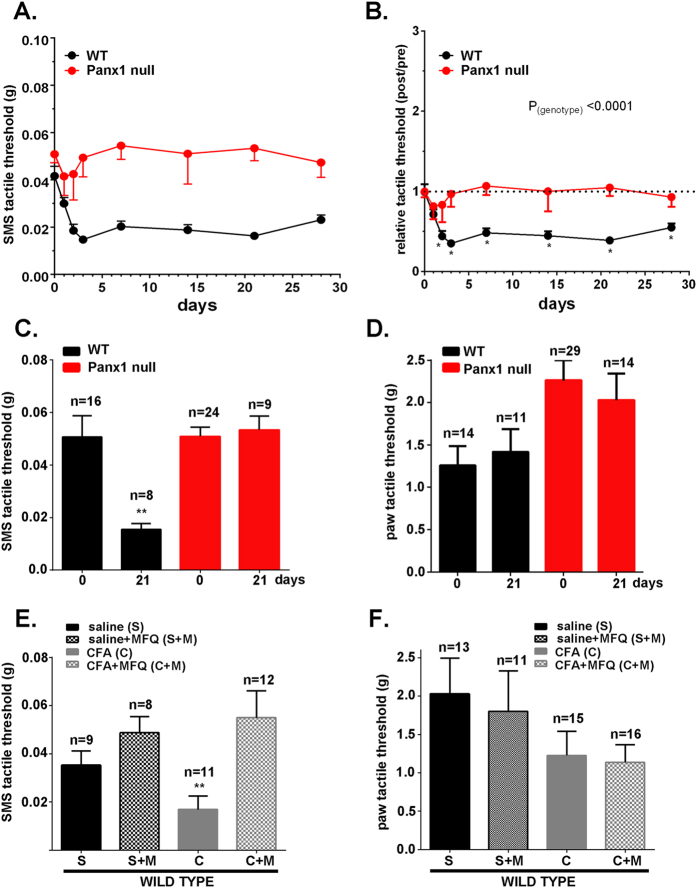
Deletion or blockade of Panx1 prevents tactile hypersensitivity in an orofacial pain model. Time course of changes of submandibular skin (SMS) tactile threshold of WT and Panx1 null mice (**A**) following CFA injection into SMS and the normalized values (post/pre-CFA injection) of SMS tactile threshold (**B**) shown in part (**A)**. Histograms of the mean ± SE values of SMS (**C**) and paw (**D**) tactile threshold obtained before (0 days) and at 21 days post-CFA injection into the SMS. (**E**,**F**) show the mean ± SE of SMS and paw tactile threshold of WT mice injected with mefloquine (MFQ) or vehicle after saline or CFA injections into SMS. (**A and B)**: n = 16-24 mice, *P < 0.05 for one-way ANOVA with repeated measures. (**C–F)**: **P < 0.001, one-way ANOVA followed by Tukeys’ multiple comparison tests; number of mice indicated above bars in histogram.

We previously described a mouse model of orofacial pain in which focal injection of CFA into the SMS induced transient inflammation and local hypersensitivity that persisted for at least seven weeks after injection^4^. When we compared WT mice to transgenic animals with global deletion of Panx1, we obtained strong evidence that Panx1 contributes to tactile hypersensitivity (Fig. 1A–C). Whereas WT mice developed allodynia rapidly (significantly different from baseline within 3 days of CFA injection) and sustained this hypersensitivity for at least 28 days thereafter, Panx1 null mice showed no change in threshold response to von Frey filaments applied to the SMS (mean tactile threshold data are shown in Fig. 1A and data normalized for each animal’s baseline response are shown in Fig. 1B). As illustrated in Supplemental Fig. 1, Panx1 heterozygous mice showed tactile thresholds that were intermediate between WT and Panx1 null mice, indicating a gene dosage effect. Tactile thresholds of Panx1 null mice before (0 days) and 21 days after CFA injection were quite similar, while thresholds of WT mice in which CFA was injected decreased significantly (Fig. 1C); hind paw tactile threshold as a substitute for an uninjected control site and measure of the overall state of sensitization was not affected by SMS injection of CFA in either genotypes (Fig. 1D). Similar results to those from Panx1 null mice were obtained from WT mice i.p. injected with the Panx1 inhibitor mefloquine (MFQ; Fig. 1E,F). MFQ treatment 2 hr before testing did not alter the sensitivity of saline-injected mice, but restored baseline threshold sensitivity in CFA-injected mice (Fig. 1E). No differences were detected in paw response thresholds (Fig. 1F).

The possibility that Panx1 null mice might have lower SMS inflammation, thereby accounting for the lack of allodynia seen in response to CFA injection in these animals was tested by comparing the number of inflammatory cells in the SMS among the genotypes. As shown in Supplemental Fig. 2, compared to saline-injected control (76.0 ± 11.4 cells/mm^2^), a significant increase in number of inflammatory cells was recorded at 7 days after CFA injection in both WT (358.1 ± 43.7 cells/mm^2^) and Panx1 null (368.1 ± 103.6 cells/mm^2^) mice; inflammation was found to be similarly resolved at 28 days in both genotypes (WT: 50.9 ± 3.2 cells/mm^2^, Panx1 null: 53.6 ± 4.4 cells/mm^2^; Suppl. Fig. 2). Because the number of inflammatory cells that infiltrated the SMS after CFA injection was not significantly different when comparing Panx1 null and WT mice, we conclude that the difference in allodynia is not attributable to differential presence of inflammatory cells. To rule out sedative effects, changes in anxiety or unusual behavior in mice caused by CFA and/or MFQ, we used the open field test battery to analyze mouse behavior in terms of general activity, exploration and anxiety. No significant behavioral differences were detected among the treatment groups (saline *vs* CFA, without or with MFQ) or between genotypes (Suppl. Fig. 3A).

### Panx1 expression and function are increased during allodynia

**Figure 2:**
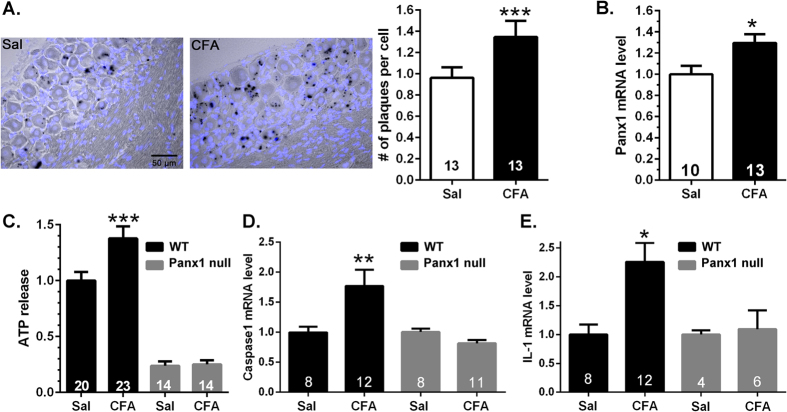
Increased Panx1 expression and function in trigeminal ganglia of CFA-injected mice. Histograms showing the mean ± SE values of the (**A**) number of LacZ plaques per cell (satellite glia and sensory neurons) and of (**B**) Panx1 mRNA levels in the trigeminal ganglia of mice 7 days after submandibular injection of CFA relative to those obtained from saline (Sal) injected ones. Numbers of mice are indicated in bars. *P < 0.05, **P < 0.01, unpaired t-test. (**C–E**) ATP release and caspase-1 and IL-1β mRNA levels are elevated in trigeminal ganglia following one week CFA injection and these changes are prevented in mice lacking Panx1. *P < 0.05, **P < 0.01, ***P < 0.001, one way ANOVA followed by Tukeys’ multiple comparison test. Numbers of mice are indicated in bars.

A potential mechanism by which Panx1 contributes to pain is by releasing the algogenic molecule ATP and in addition activating the production of cytokines. Using LacZ (which in the Panx1 null mouse is inserted into the Panx1 gene locus) as a surrogate marker for the activation of Panx1 expression, we show that in the trigeminal ganglia of Panx1 null mice, there is significantly higher LacZ staining in neurons and satellite glial cells from mice treated with CFA compared to saline-injected ones (about 40% more LacZ positive plaques: Fig. 2A). Evidence that Panx1 transcripts are increased in our pain model was also provided by quantifying Panx1 mRNA levels in trigeminal ganglia from saline and CFA-injected WT mice (revealing a difference of about 30%: Fig. 2B). We also found that the activity of Panx1 channels was enhanced during allodynia; there was significantly higher ATP released from trigeminal ganglia of CFA-injected compared to saline-injected WT mice (Fig. 2C). ATP released from trigeminal ganglia of Panx1 null mice, in contrast, did not differ in the saline- and CFA-injected groups, and the amount of ATP released in both Panx1 null groups was much lower than that measured from WT ganglia (Fig. 2C). Moreover, we also found that WT but not Panx1 null mice injected with CFA exhibited substantially higher levels of caspase-1 and IL-1β mRNA (Fig. 2D,E). These data indicate that increased expression of Panx1 transcript positively correlates with increased channel function and cytokine production.

### Increased activity in satellite glial cells and sensory neurons in trigeminal ganglia of CFA-treated mice

**Figure 3:**
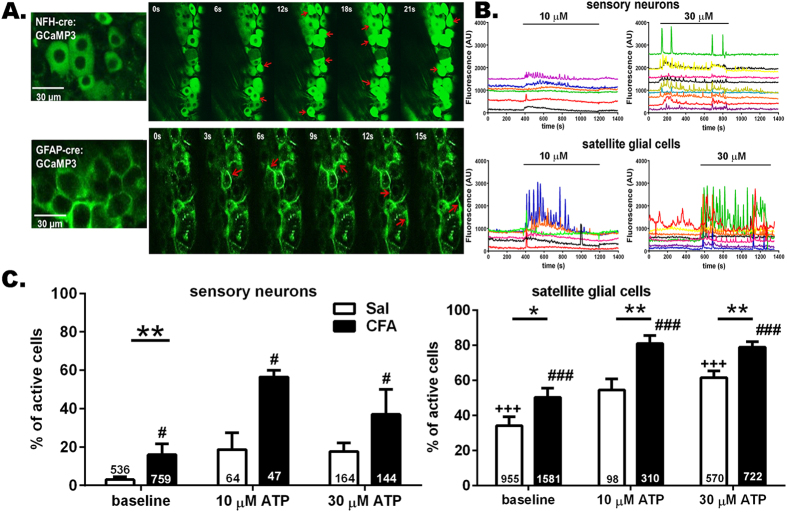
ATP enhances hyperactivity of trigeminal ganglion neurons and satellite glia of mice with orofacial pain. (**A**) Confocal images showing GCaMP3 fluorescence changes in intact trigeminal ganglia reporting Ca^2+^ dynamics in neurons with NFH-Cre:GCaMP3 expression and satellite glial cells with GFAP-Cre:GCaMP3 expression. Red arrows indicate representative neurons and satellite glial cells showing Ca^2+^ elevations during this short (21 and 15 sec in upper and lower panels) recording epochs. (**B**) ATP stimulation of trigeminal ganglia increased intracellular Ca^2+^ concentration in sensory neurons and satellite glial cells. Representative traces of Ca^2+^ transients recorded from neurons and satellite glial cells to increasing concentrations of ATP (10 and 30 μM). (**C**) The proportion of active neurons and satellite glial cells at baseline is significantly higher in trigeminal ganglia from CFA-injected than in saline-injected mice; application of ATP increased the proportion of active neurons and satellite glial cells in trigeminal ganglia derived from one week CFA-treated mice (baseline *vs* ATP). *P < 0.05, **P < 0.01, one way ANOVA (saline *vs* CFA). ^**+++**^P < 0.005, ^**#**^P < 0.05 and ^**###**^P < 0.001, one way ANOVA followed by Sidak’s multiple comparison test for ATP doses in saline and CFA groups, respectively. The total number of cells analyzed derived from 4-15 mice are indicated in the bars.

To evaluate the extent to which cellular activity within the trigeminal ganglia was modified in our model of orofacial pain and the extent to which ATP modulates the activity, we measured Ca^2+^ transients in trigeminal ganglia of mice in which expression of the Ca^2+^ indicator GCaMP3 was targeted to GFAP-positive glia or to neurons. As shown in Fig. 3A and B, both sensory neurons (NFH-Cre:GCaMP3) and satellite glial cells (GFAP-Cre:GCaMP3) from saline- and CFA-injected mice exhibited spontaneous ongoing Ca^2+^ transients and responded with augmented Ca^2+^ transients to bath application of ATP. In CFA-injected mice, the number of sensory neurons and satellite glia cells displaying spontaneous Ca^2+^ transients was significantly higher compared to those recorded from saline-injected mice (Fig. 3C; baseline). Bath application of ATP further increased the proportion of active cells within the trigeminal ganglia of saline- and CFA-injected mice (Fig. 3C). Although higher ATP concentrations were tested (see data for 30 μM in Fig. 3B,C), the maximal recruitment of active cells was achieved at 10 μM ATP and did not further change with higher dose of ATP (Fig. 3C). With regard to the frequency of Ca^2+^ transients, in both sensory neuron and satellite glial cells there was significantly higher baseline frequency of Ca^2+^ spikes in trigeminal ganglia of CFA-injected compared to saline-injected mice (Suppl. Fig. 4). Bath application of ATP (10 μM but not 30 μM) increased the frequency of Ca^2+^ transients in satellite glia but not in sensory neurons (Suppl. Fig. 4).

### Targeted deletion of Panx1 in GFAP-positive glia or neurons differentially affects the time course of tactile hypersensitivity

**Figure 4:**
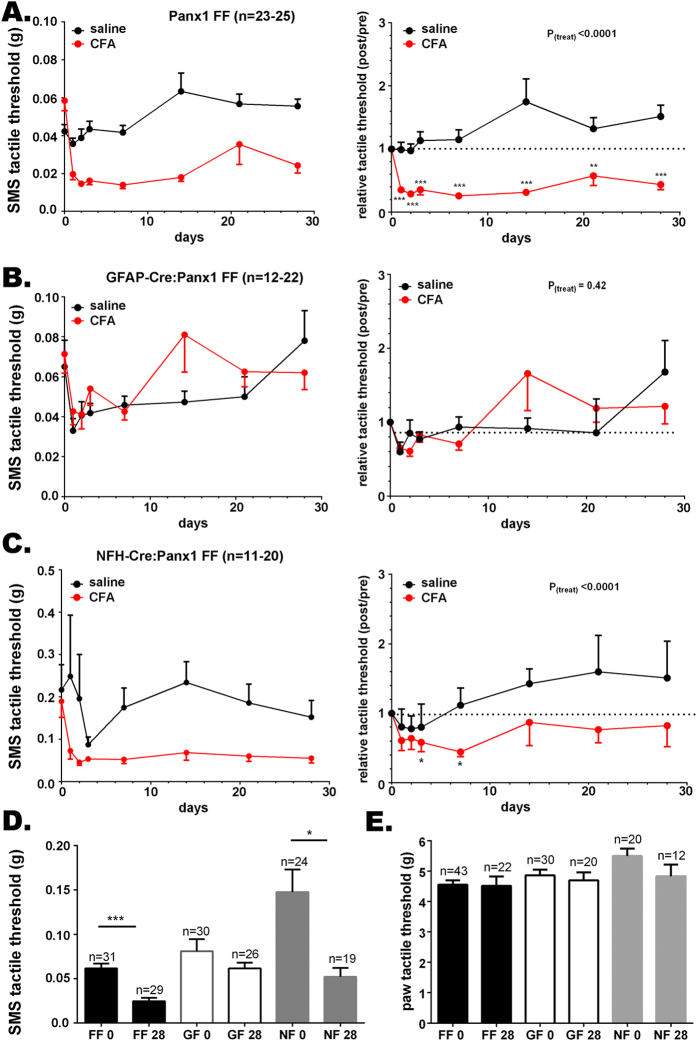
Panx1 deletion in GFAP-positive glia and neurons differentially affects tactile hypersensitivity. Time course of changes of submandibular skin (SMS) tactile threshold of (**A** left) Panx1^fl/fl^, (**B** left) GFAP-Cre:Panx1^fl/fl^, and (**C** left) NFH-Cre:Panx1^fl/fl^ following CFA injection into the submandibular skin; (**A–C** right) are the normalized values (post/pre-CFA injection) of SMS tactile threshold shown in part **A**. Histograms of the means ± SE values of SMS (**D**) and hind paw (**E**) tactile threshold obtained before (0 days) and at 28 days post-CFA injection into the submandibular skin. Parts **A–C**: *P < 0.05, **P < 0.01, ***P < 0.001 from one-way ANOVA with repeated measures. Parts **D-E**: *P < 0.05, ***P < 0.001, One-way ANOVA followed by Tukeys’ multiple comparison tests. Number of mice indicated. FF: Panx1^fl/fl^, GF: GFAP-Cre:Panx1^fl/fl^; NF: NFH-Cre:Panx1^fl/fl^; 0 and 28 indexes: before and 28 days after CFA injection, respectively.

Panx1 channels are present in both neurons and satellite glial cells in trigeminal ganglia of mice^10^ (Suppl. Fig. 5). To determine the contribution of GFAP-positive glia and neuronal Panx1 to orofacial pain, we tested the tactile sensitivity in animals in which Panx1 was deleted selectively in neurons and GFAP-positive glial cells using the NFH-Cre:Panx1^fl/fl^ and GFAP-Cre:Panx1^fl/fl^ mice, respectively. As shown in Fig. 4, mice with the GFAP-targeted Panx1 deletion did not develop hypersensitivity after CFA injection (Fig. 4B), whereas their Panx1^fl/fl^ control littermates displayed tactile hypersensitivity (Fig. 4A; raw data are shown in left hand panels and data normalized to baseline responses are shown on the right). As for the global Panx1 null mice (Fig. 1), the absence of tactile hypersensitivity in mice lacking Panx1 in GFAP-positive glial cells was unrelated to the degree of inflammation induced by submandibular injection of CFA. The number of inflammatory cells that infiltrated the SMS after CFA injection was not significantly different from that seen in Panx1^fl/fl^ or WT mice (Suppl. Fig. 2). Compared to saline-injected controls (76.0 ± 11.4 cell per mm^2^), a significantly higher inflammatory cell infiltration was recorded at 7 days after CFA injection in both Panx1^fl/fl^ (495.9 ± 98.8 cells/mm^2^) and GFAP-Cre:Panx1^f/fl^ (383.1 ± 46.7 cells/mm^2^) mice; inflammation was found to be resolved at 28 days after CFA in both genotypes (Suppl. Fig. 2). SMS tactile thresholds of GFAP-Cre:Panx1^fl/fl^ mice before (0 days) and 28 days after CFA injection were not significantly different, while those of the Panx1^fl/fl^ controls were significantly lower (Fig. 4D); paw tactile threshold was not affected by SMS injection of CFA in either genotype (Fig. 4E).

**Figure 5:**
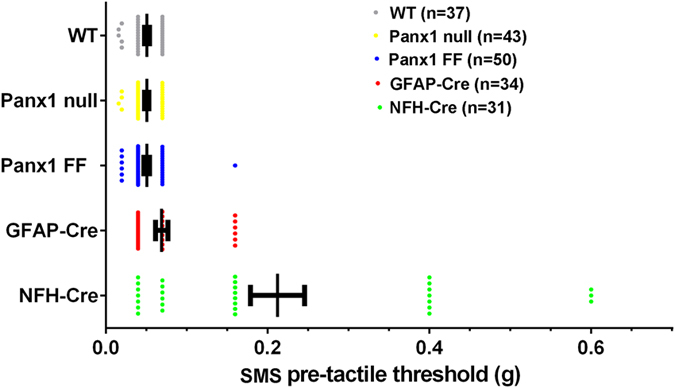
Comparison of basal SMS tactile thresholds in all the genotypes studied here. Tactile sensitivity was assessed before injection of saline or CFA using von Frey filaments to determine baseline tactile sensitivity. Each point represents SMS tactile threshold obtained for each mouse measured with von Frey filaments before injection of either saline or CFA, and the range bars represent mean ± SE values of SMS tactile threshold obtained for each genotype. Note that mice with NFH-Cre targeted deletion show higher mean and a wider range of variability in tactile threshold values. WT: wild type, Panx1 null, Panx1 FF: Panx1^fl/fl^, GFAP-Cre: GFAP-Cre:Panx1^fl/fl^, NFH: NFH-Cre:Panx1^fl/fl^. n = number of animals.

In contrast to mice with glia-targeted deletion of Panx1, the CFA-induced tactile hypersensitivity in NFH-Cre:Panx1^fl/fl^ mice was transient (Fig. 4C). Two weeks after CFA injection, no significant differences in threshold were detected compared to Panx1^fl/fl^ control mice. At baseline, the SMS tactile threshold of mice with Panx1 deleted from neurons was about three times higher than the mean threshold values of Panx1^fl/fl^ and GFAP-Cre:Panx1^fl/fl^ (Fig. 4D); paw tactile threshold at baseline was also significantly higher than the average (Fig. 4E). Figure 5 summarizes the baseline SMS tactile thresholds for all mouse genotypes; the baseline SMS tactile threshold of WT, global Panx1 deletion and the Panx1^fl/fl^, were virtually identical. In the GFAP-specific deletion, there were a few mice with higher thresholds than the first three groups, although the overall difference was not significant. In the NFH-targeted deletion group, however, the majority of mice showed higher mean values and large variability.

The degree of SMS inflammation induced by CFA injections at 7 days and its resolution at 28 days were similar to those recorded for all other genotypes (Suppl. Fig. 2). Behavioral analyses using the open field test battery revealed that the distances traveled and the number of rears did not differ between the treated groups (saline *vs* CFA) or among genotypes (Panx1^fl/fl^, GFAP-Cre:Panx1^fl/fl^ and NFH-Cre:Panx1^fl/fl^; Suppl. Fig. 3B). However, both saline- and CFA-injected NFH-Cre:Panx1^fl/fl^ mice made fewer visits to the center of the open field than Panx1^fl/fl^; the other genotypes did not differ (Panx1^fl/fl^ and GFAP-Cre:Panx1^fl/fl^; Suppl. Fig. 3B).

In terms of ATP released from trigeminal ganglia, we found that at one week after CFA injection, a time point in which mice from both cell-specific knockouts show decreased pain sensitivity compared to control (Panx1^fl/fl^) mice (Fig. 4), ganglia from CFA-injected Panx1^fl/fl^ mice showed higher ATP release than saline injected Panx1^fl/fl^ mice; such differential ATP release in CFA-injected mice was not seen in either GFAP- or NFH-targeted Panx1 deleted ganglia (Supplemental Figure 6).

Results from these experiments not only indicate that Panx1 plays an important role in the development of allodynia but also disclose the differential contribution of GFAP-positive glia and neurons to the baseline threshold and persistence of hypersensitivity/allodynia.

## Discussion

Here we provide the first direct evidence that Panx1 plays a crucial role in the development of allodynia following CFA injection into the submandibular skin (SMS), a readily quantifiable orofacial pain model that we had previously described^4^. Using transgenic mice with global or cell-type specific deletion of Panx1, we show that tactile hypersensitivity of the injected area is totally abolished in mice lacking Panx1 as well as in mice with Panx1 deleted from GFAP-positive glial cells. Thus, Panx1, particularly in GFAP-positive glial cells, is a potentially powerful new target for the development of non-narcotic pain medication.

Panx1 is a member of the gap junction family of proteins that forms plasma membrane channels which are opened under physio-pathological conditions by stimuli such as ligand binding to several types of transmitter receptors (in particular the P2x7, but also P2x4, glutamate and adrenergic receptors), membrane stretch and moderately elevated concentrations of extracellular K^+^ (see^19^). Upon activation, Panx1 channels release ATP and associate with inflammasome components leading to caspase-1 cleavage and the production of IL-1β^12^,^20^. Panx1 is found both in glia and neurons in the CNS and PNS^9^,^10^,^16^,^21^ and its activation has been shown to contribute to the development of several disorders, including migraine^17^ and neuropathic pain^14^,^15^,^16^. However, the evidence that Panx1 contributes to pain has relied on use of pharmacological agents, almost all of which act on channels formed of other gap junction proteins, the connexins. Indeed, expression of Cx43 and gap junction mediated coupling among satellite glial cells in trigeminal and dorsal root ganglia were shown to be increased in several pain models and pharmacological blockade and/or downregulation of gap junction channels was found to attenuate mechanical and/or thermal hypersensitivity^4^,^22^,^23^,^24^,^25^,^26^.

In neuropathic and inflammatory pain conditions involving the trigeminal ganglia, alterations have been reported in expression levels of molecules involved in thermal and tactile hypersensitivity/hyperalgesia such as the purinergic P2x3 receptors in sensory neurons^27^ and of the pro-inflammatory cytokine, IL-1β, in satellite glial cells^25^,^28^. Here we show that Panx1 mRNA levels are increased in trigeminal ganglia after CFA injection into the SMS, and this increase is associated with higher caspase-1 and IL-1β mRNA levels and augmented ATP release. These molecular changes, which are concurrent with the development of SMS tactile hypersensitivity, were found to be blunted in CFA-treated mice in which Panx1 is deleted. Similarly, Zhang *et al*.^16^ reported that Panx1 was upregulated in the rat dorsal root ganglia after nerve injury and that knockdown of Panx1 by intra-thecal injection of Panx1 siRNA not only attenuated tactile hyperalgesia but also diminished the levels of caspase-1, the enzyme that cleaves pro-IL-1β to its mature form. That Panx1 induces expression of P2 receptors and of inflammation-related genes indicates that it can be regarded as a major contributor to pain by enhancing both purinergic and cytokine signaling.

Evidence that ATP is involved in pain responses includes (i) the induction of pain when skin or nerve are exposed to ATP, (ii) detection of elevated ATP levels in sensory ganglia and spinal cord in experimental pain models such as spared nerve ligation, and (iii) ATP induced activation of purinergic receptors linked to cytokine production^29^. In particular, activation of Panx1 through the P2x7^12^,^30^, P2x3^31^, and P2x4^32^ receptors have been shown to lead to maturation and release of pro-inflammatory mediators, thereby sensitizing peripheral and spinal cord neurons^31^,^32^. We found that behavioral hypersensitivity in our orofacial pain model is accompanied by increased Panx1 expression and that neurons and GFAP-positive glia in the trigeminal ganglia become hyper-excitable. The number of sensory neurons and satellite glial cells displaying Ca^2+^ transients as well as the frequency of their activity were elevated in trigeminal ganglia of CFA- compared to saline-treated mice, and bath application of ATP to trigeminal ganglia potentiated the differences in activity between ganglia from CFA-treated and control mice. We thus hypothesize that augmentation of Panx1 function in response to painful stimuli contributes to hyper-excitability in the trigeminal ganglia, resulting in allodynia.

Glial activation and neuronal-glia interaction are mechanisms involved in nociception not only in the central nervous system (CNS), but also in the periphery^33^. Given that both sensory neurons and satellite glial cells express Panx1^10^,^16^,^21^ (see Suppl. Fig. 4), we assessed the relative contribution of these two cell types to tactile hypersensitivity using mice in which Panx1 was selectively deleted in neurons or GFAP-positive glia. Behavioral analyses showed that Panx1 in GFAP-positive glial cells is critical for the development and maintenance of tactile hypersensitivity and that while neuronal Panx1 only contributes to the initial, acute stage of hypersensitivity, it greatly influences basal tactile threshold. The critical role played by GFAP-positive glial Panx1 is likely related to cytokine production, which is prominent in satellite glial cells after injury^34^,^35^,^36^,^37^. Not only is Panx1 directly associated with inflammasome components leading to IL-1β release but also indirectly through its association with the P2x7 receptors, which are expressed in satellite glial cells^38^,^39^,^40^. In addition, given the close topological proximity between satellite glial cells and sensory neuron cell bodies, efficient activation of neuronal P2x3 receptors by ATP released through Panx1 channels may be amplified by the excitatory effects of the satellite glial cytokine resultant from its activation. The transient tactile hypersensitivity seen in NFH-Cre:Panx1^fl/fl^ mice is likely related to a mechanism in which ATP is normally released spontaneously from neurons, causing persistent activation of satellite glial cells. In the absence of neuronal Panx1, ATP would only be released from neurons via synaptic vesicles located at distances too far from the ganglia to be sufficient to activate the satellite glial cells. In this sense, neuronal Panx1 would provide a positive feedback loop that, through neuron-glia interaction would continuously activate the satellite glia.

The higher baseline tactile threshold in mice with Panx1 deleted from neurons is surprising and unexpected; elevated threshold was not restricted to the SMS but also found in the paw. The possibility that genetic background contributed to differences in tactile threshold observed in NFH-Cre:Panx1^fl/fl^ compared to other transgenic mice used here seems unlikely. Although the NFH-Cre mice originally purchased from Jackson are reported to have a mixed background (129/Sv * 129 S7/SvEvBrd * C57BL/6 * FVB), these mice were backcrossed with Panx1^fl/fl^ (C57Bl/6) for at least four generations before use, which further diluted the potential genetic influence imposed by the mixed background. In addition, it was previously reported that the tactile thresholds of inbred C57Bl6, 129/SV and FVB mice are not different^41^. Alternatively, the increased threshold of the NFH-Cre:Panx1^fl/fl^ mice could arise from the absence of ATP leakage from neurons through Panx1 channels which normally sensitized the neurons; thus in mice with neuronal deletion of Panx1, the absence of this release mechanism would relieve the ATP-induced neuronal depolarization rendering them more hyperpolarized. Alternatively and /or in addition, the higher tactile threshold in the NFHCrePanx1^fl/fl^ mice may be a supraspinal consequence of the deletion, resulting in distinct somatotopic organization, cognitive performance and anxiety. These are more likely scenarios given that the targeted deletion is not restricted to sensory neurons, but affect CNS areas involved in pain perception.

In summary, our findings show that CFA-induced peripheral inflammation increases Panx1 levels in trigeminal ganglia leading to enhanced purinergic signaling and cytokine production. Enhanced Panx1 expression and function most likely contribute to the maintenance of a hyper-excitable state. In addition, we show that GFAP-positive glial and neuronal Panx1 differentially contribute to the development and maintenance of persistent tactile hypersensitivity.

## Methods

### Trangenic mice

Mice with global and cell type specific deletion of Panx1 used in the present study were generated as previously described^10^. Briefly, Panx1-null mouse line (Panx1^tm1a(KOMP)Wtsi^) generated by the Knock Out Mouse Project, KOMP (www.KOMP.org) in the C57BL/6 background were maintained in our animal facility at Albert Einstein College of Medicine as global Panx1 knockout (Panx1 null: Panx1^−/−^) and wild-type (WT: Panx1^+/+^). Panx1^fl/fl^ mice were generated in our facility from crosses between Panx1^tm1a(KOMP)Wtsi^ and a flippase deleter mice (B6.ACTFLPe/J) in the C57BL/6 background. For GFAP-positive glial and neuronal deletion of Panx1, mGFAP-Cre (B6.Cg-Tg(Gfap-cre)73.12 Mvs/J) in the C57BL/6 background and mNFH-Cre (Tg(Nefh-cre)12 Kul/J) mice in a mixed (129/Sv * 129S7/SvEvBrd * C57BL/6 * FVB) background purchased from Jackson laboratory were crossed with Panx1^fl/fl^ to generate mGFAP-Cre:Panx1^fl/fl^ and mNFH-Cre:Panx1^fl/fl^ mice which were maintained in our animal facility.

Mice with targeted expression of a calcium indicator (GCaMP3; B6;129S-Gt(ROSA)26Sortm38(CAG-GCaMP3)Hze/J in the C57BL6 background) were purchased from Jackson laboratory and crossed with either the mGFAP-Cre (B6.Cg-Tg(Gfap-cre)73.12 Mvs/J) or the mNFH-Cre (Tg(Nefh-cre)12 Kul/J) mice to obtain offspring expressing GCaMP3 in GFAP-positive glia and neurons, respectively.

For all experiments described below we used 2–4 month old male and female mice. All animal procedures were conducted in accordance with the Policies on Animal Research of Albert Einstein College of Medicine and were approved by the Institutional Animal Care and Use Committee of Albert Einstein College of Medicine (Protocol number 20151101).

### Tactile sensitivity and Open field analyses

Assessment of tactile sensitivity was performed as previously described^4^. Mice were briefly anesthetized with isoflurane to inject saline or Complete Freund’s Adjuvant (CFA) into the midline of the submandibular skin (SMS), which induced hypersensitivity to von Frey filament testing beginning about 3 days later. Tactile sensitivity of the SMS was quantitatively assessed using calibrated von Frey filaments (Stoelting, Wood Dale, IL, USA). Ascending force intensities of von Frey filaments were applied to the SMS to determine the minimum force that evoked a withdrawal response. Each filament was applied 10 times with 5–30 s random intervals to determine tactile threshold which was defined as the minimal force that induced a withdrawal response in 8 of 10 trials. Tactile responses were assessed before and on 1–4 weeks post CFA or saline injection. The effects of a Panx1 channel blocker, mefloquine (MFQ, Bioblocks #QU024-1; 10 mg/kg body weight, i.p. injected 2 hours before behavior testing) were investigated at 1, 3, and 4 weeks after CFA injection in wildtype (WT: Panx1^+/+^) mice.

We also performed a full open field analysis (6 min total test) analyzed with Viewer automated tracking software (Biobserve; Bonn, Germany). The behavioral assessments included locomotor activity (distance traveled), and exploration (number of rears) and anxiety (number of entries to center). In all behavioral tests, testers were blind to the condition of the mice.

### Skin inflammation scoring

Inflammation in the SMS of mice was assessed before and at 1–4 weeks after subcutaneous CFA or saline injection. For that, mice were sacrificed after isoflurane anesthesia; the SMS was removed and fixed overnight in 4% p-formaldehyde (PFA) in PBS, pH 7.4 at 4 °C. The skin tissues were kept in 70% ethanol until paraffin embedding. The degree of skin inflammation was determined by counting the number of inflammatory cells present on the tissue using hematoxylin-eosin (H&E) staining of 5 μm-thick sections. For morphological analysis, every 4^th^ section stained with H&E was examined by microscopy to compute the number of white blood cells/mm^2^ averaged over a total of 6–10 sections.

### LacZ staining

In the Panx1 null mouse used for these studies, LacZ (encoding beta-galactosidase) is expressed under the control of the endogenous reporter, allowing direct analysis of promoter activity. Trigeminal ganglia were fixed for 1 hour in 4% PFA in PBS pH 7.4, rinsed multiple times and washed for at least 30 min with wash buffer [2.0 mM MgCl_2_, 0.01%deoxycholate, 0.02% NP-40, 97 mM Na_2_PO_4_, pH 7.3] at RT, and then incubated in a X-gal reaction solution (1.0 g/l X-gal, 5.0 mM potassium ferrocyanide, 7.0 mM potassium ferricyanide) overnight at 37 °C. Tissues were then rinsed with wash buffer and post-fixed overnight in 4% PFA at 4 °C. Samples were incubated in sucrose solution (10, 20, 30% sucrose in PBS for 24 hours each at 4 °C), frozen in O.C.T. and cut in 10 μm thin sections. Tissue sections were stained with 0.5 μg/ml 4′,6-diamidino-2-phenylindole (DAPI)/PBS for 1 hour, rinsed with PBS pH 7.4 for 3 × 15 min and counterstained with nuclear fast red from Vector labs (H-3403) for 1 min. Photomicrographs of tissue sections were captured using a Leica DFC360 FX (fluorescence) and DFC 490 camera mounted on a Leica DM6000 microscope. Images of tissue sections were acquired and analyzed using Leica Application Suite and Leica Application Suite FX softwares. All raw data was imported into Adobe Photoshop CS5 and corrected for brightness and contrast levels only. Number of LacZ positive plaques per cell (satellite glia and sensory neurons) was counted in 5–10 tissue sections per mouse ganglion.

### Real-time RT-PCR

Real-time RT-PCR was performed using SYBR Green PCR Master Mix (Applied Biosystems, Life Technologies) with 7300 Fast Real-Time PCR system (Applied Biosystems, Life Technologies). Complementary DNA was synthesized from 1 μg of RNA extracted from whole trigeminal ganglia of mice one week after saline or CFA injection using a Superscript VILO cDNA Synthesis Kit (Invitrogen, Life Technologies, Grand Island, NY, USA). Reaction mixtures were denatured at 95 °C for 10 min, followed by 40 PCR cycles. Each cycle consisted of following three steps: 94 °C for 15 s, 57 °C for 15 s, and 72 °C for 1 min. Each sample was normalized against an internal 18 s ribosomal RNA control. Primers used were designed using Primer Express 2.0 software (Applied Biosystems) and are listed below.

Panx1 (F: AGCCAGAGAGTGGAGTTCAAAGA, R: CATTAGCAGGACGGATTCAGAA).

IL-1β (F: CAGGCAGGCAGTATCACTCA, R: TGTCCTCATCCTGGAAGGTC).

Caspase1(F: GCTTCAATCAGCTCCATCAGC, R: GACGTGTACGAGTGGTTGTATTCA).

18 s (F: CACGGCCGGTACAGTGAAAC, R: AGAGGAGCGAGCGACCAAA).

### ATP release

The amount of ATP released from whole trigeminal ganglia of mice one week after saline or CFA injection was determined as previously described^9^ using the luciferin/luciferase assay (Molecular Probes) and a Turner luminometer. Trigeminal ganglia were excised and immediately placed into ice cold (0-4 °C) HEPES buffered, air bubbled artificial cerebrospinal fluid (ACSF: 145 mM NaCl, 2.5 mM KCl, 3.1 mM CaCl_2_, 1.3 mM MgCl_2_, 10 mM glucose, 10 mM Hepes, pH 7.2). Dissected ganglia were then placed in MatTeck dishes containing 200 μl ACSF, at 37 °C 45 min and aliquots of ACSF solution collected after incubation period and stored at −20 °C until use. The concentration of ATP released by trigeminal ganglia into the ACSF was obtained from standard curves and values normalized to the total amount of protein. Total protein concentration was determined from trigeminal ganglion homogenates (lysis buffer: 150 mM NaCl; 10 mM Tris-base; 1% TritonX-100; pH 7.4) using the BCA reagents (Thermo Fisher).

### Calcium imaging

Trigeminal ganglia excised from mice expressing the Ca^2+^ indicator GCaMP3 in neurons or glia were evaluated. Ca^2+^ transients were recorded from trigeminal ganglia using a confocal laser scanning microscope, Olympus Fluoview 300 equipped with 40× water-immersion lens (0.80 NA) and camera, filter set (emission/ excitation), software. Images were acquired at a rate of 40 frames/min. Excitation light was provided by an argon (488 nm) laser (Melles Girot, Carlsbad, CA). Ca^2+^ transients were quantified and expressed as the percentage of active cells within the field of view and as the frequency of Ca^2+^ spikes per min using ImageJ on selected regions placed on satellite glia and neurons of trigeminal ganglia derived from saline- and CFA-injected mice. Similar analyses were performed on ganglia exposed to ATP (10 and 30 μM).

### Immunohistochemistry

Detection of Panx1 in satellite glia and neurons from trigeminal ganglia was performed as previously described^10^ using chicken anti-Panx1 (1:500; extracellular loop (CZVQQKSSLQSES); Aves Lab #6358); mouse anti-NeuN (1:100; Millipore - MAB377); goat anti-glutamine synthase (1:200; Santa Cruz - sc-6640), and secondary Alexa conjugated goat anti-chicken, goat anti-mouse, and donkey anti-goat antibodies (1:2000). Images were acquired using an Olympus confocal laser scanning microscope equipped with a 40× water-immersion lens (0.80 NA), and FITC, Texas red and UV filter sets.

### Statistical Analyses

Data are expressed as mean ± SE. Statistical analyses were conducted using GraphPad Prism 6 software. One way ANOVA followed by Tukey’ and Sidak’ multiple comparison test, and t-test were used when appropriate. Significance level set at P < 0.05.

## Additional Information

**How to cite this article**: Hanstein, R. *et al*. Glial pannexin1 contributes to tactile hypersensitivity in a mouse model of orofacial pain. *Sci. Rep.* **6**, 38266; doi: 10.1038/srep38266 (2016).

**Publisher's note:** Springer Nature remains neutral with regard to jurisdictional claims in published maps and institutional affiliations.

## Supplementary Material

Supplementary Information
