# Supplementary material for: Glial pannexin1 contributes to tactile hypersensitivity in a mouse model of orofacial pain
Source: Sci Rep. 2016 Dec 2;6:38266. doi: 10.1038/srep38266 (PMC5133615; doi:10.1038/srep38266)

Glial pannexin1 contributes to tactile hypersensitivity in a mouse model of orofacial pain

*Regina Hanstein[1], Menachem Hanani[2], Eliana Scemes[1], David C. Spray[1]**

[1]Dominick P. Purpura Department of Neuroscience, Albert Einstein College of Medicine, Bronx, NY, 10461, USA

[2]Laboratory of Experimental Surgery, Department of Surgery, Hadassah University Hospital, Jerusalem, Israel

Supplemental Figure 1. Time course of the mean ± SE normalized (post-/pre-CFA injection) changes of submandibular skin tactile threshold of CFA-treated Panx1$^{+/+}$, Panx1$^{+/-}$ and Panx1$^{-/-}$ mice. Numbers of animals indicated in parentheses.

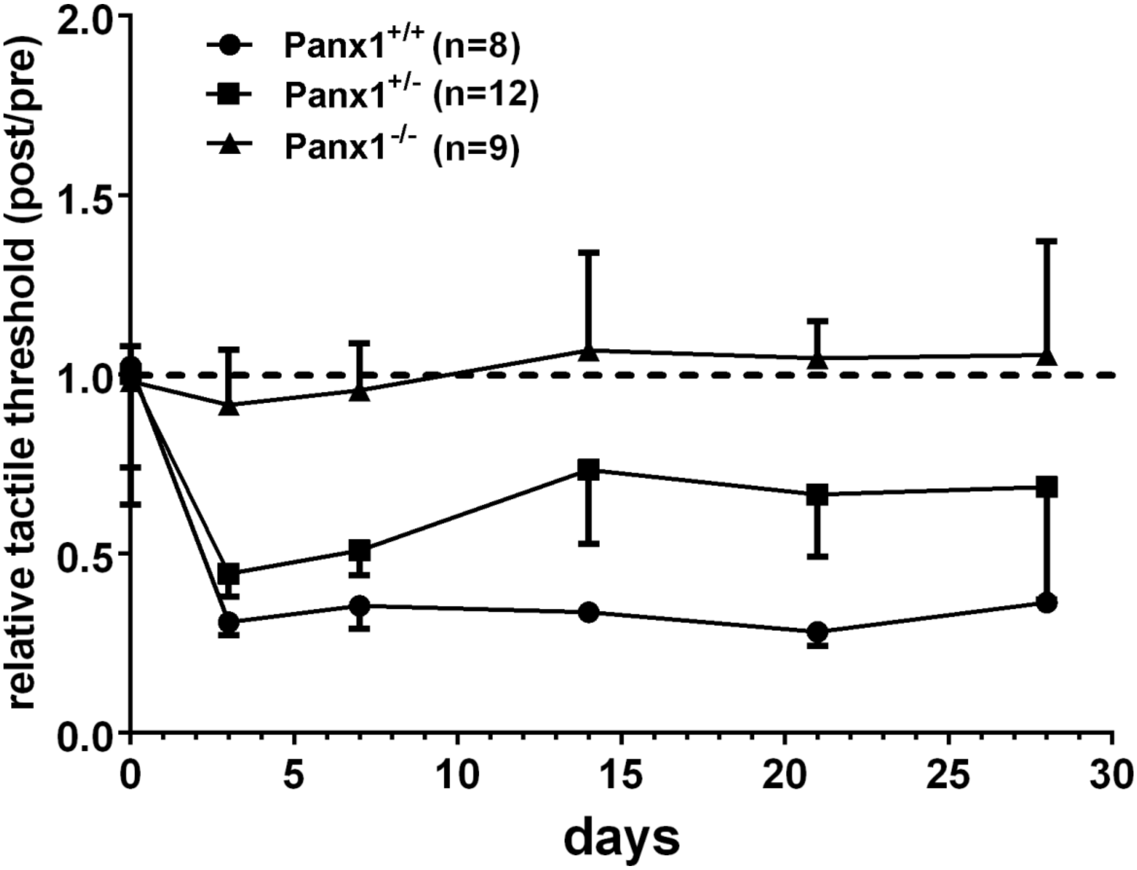

Supplemental Figure 2. Number of inflammatory cells per mm^2 recorded in the submandibular skin after subcutaneous injection of saline and CFA. (**left**) Examples of H&E stained tissues of saline- or CFA-injected WT and Panx1 null mice showing inflammatory cells (dark nuclei). (**right**) Mean ± SE values on inflammatory cells per mm^2 of submandibular skin tissues. No significant differences (P>0.05, ANOVA) were detected among the genotypes at 7 days post-injection and between the control saline-injected and 28 days post-CFA injection among the different genotypes. Numbers of animals are indicated on the top of each bar. CRTL: saline control, WT: wild type, Panx1 null, Panx1FF: $Panx1^{fl/fl}$, GFAP-Cre: GFAP-Cre:$Panx1^{fl/fl}$, NFH-Cre: NFH-Cre:$Panx1^{fl/fl}$.

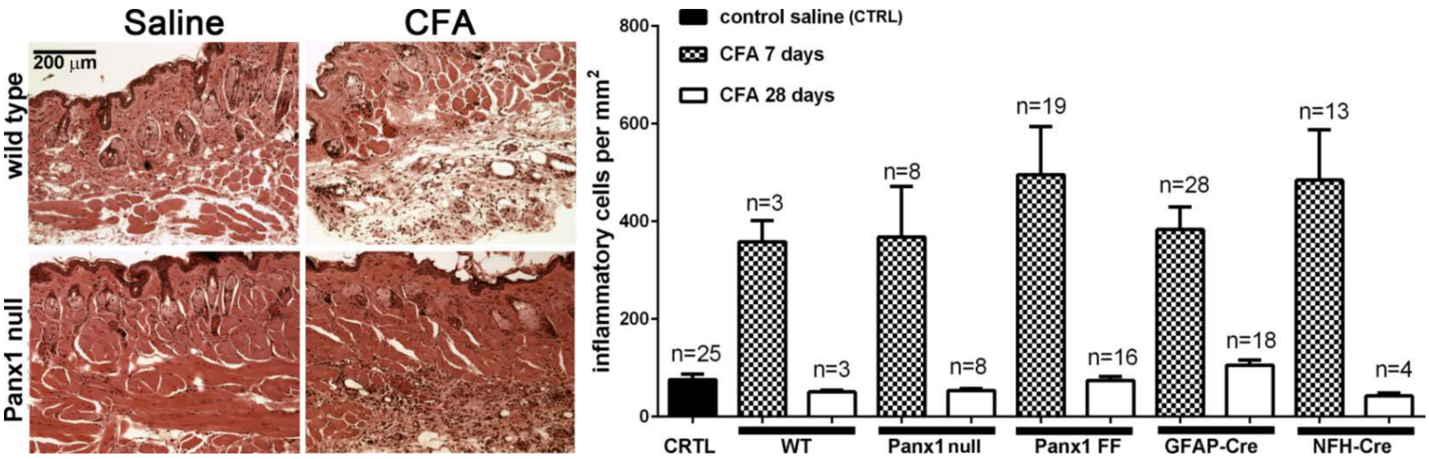

Supplemental Figure 3. Open field behavioral analyses showing the mean ± SE values of the distance traveled (activity), number of rears (exploration) and visits to the center of the field (anxiety) recorded from (A) untreated wild type - WT and Panx1$^{-/-}$ mice and from WT mice after 7 days of saline (sal) and CFA injection into the submandibular skin untreated and 2 hours after i.p. injection of mefloquine (MFQ) or vehicle. Number of animals: 6 - 35.

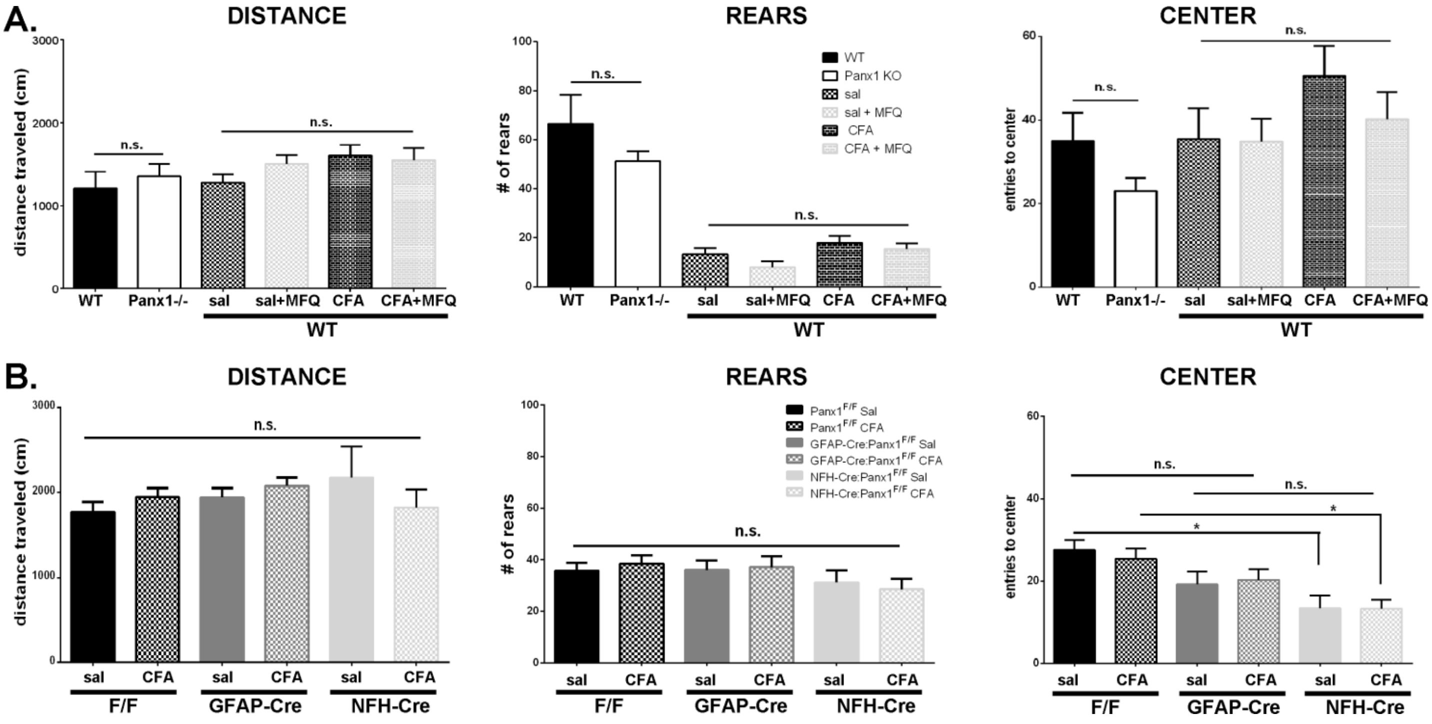

Supplemental Figure 4. Frequency of Ca^{2+} transients (spikes/min) recorded from sensory neurons (NFH-Cre:GCaMP3) and satellite glial cells (GFAP-Cre:GCaMP3) at baseline and after addition of two doses of ATP (10 and 30 µM). Bars correspond to mean ± SE values measured from active cells (number in bars). *P<0.05, **P<0.01, one way ANOVA (saline *vs* CFA) followed by Sidak's multicomparison test. Symbols $^{+}$ and $^{\#}$ P<0.05, one way ANOVA followed by Sidak's multiple comparison test for ATP doses in saline and CFA groups, respectively. Numbers of active cells derived from 4-17 mice are indicated in the bars.

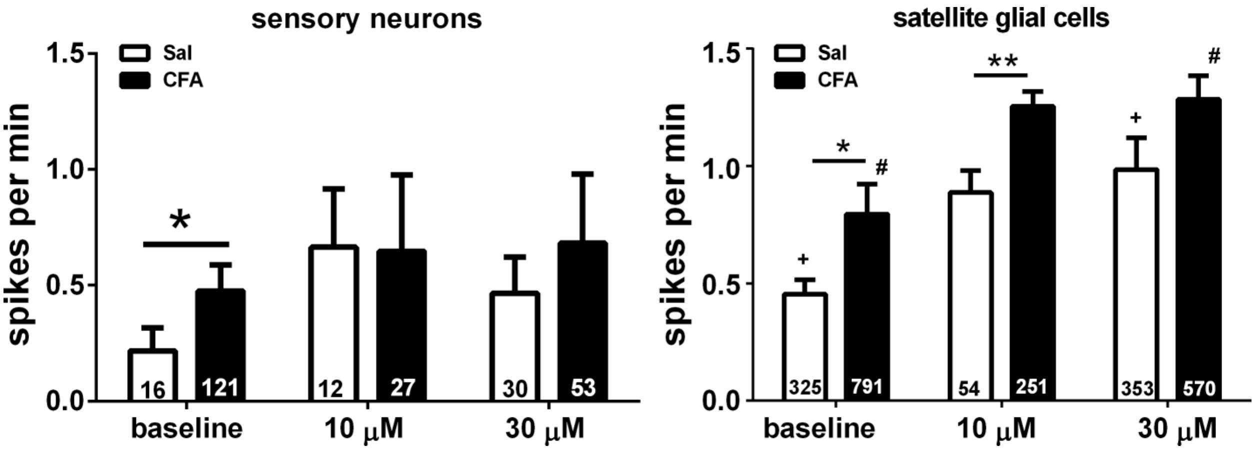

Supplemental Figure 5. *Panx1 expression in trigeminal ganglia*. Confocal images of trigeminal ganglia derived from (**A**) Panx1$^{fl/fl}$, (**B**) NFH-Cre:Panx1$^{fl/fl}$, and (**C**) GFAP-Cre:Panx1$^{fl/fl}$ mice showing expression of Panx1 (red) in neurons (arrowheads) and satellite glial cells (arrows). NeuN and glutamine synthase (GS) antibodies (green) were used to identify neurons and satellite glial cells, respectively. (**A**) Image of trigeminal ganglion of Panx1$^{fl/fl}$ mice immunostained with anti-NeuN (green) and anti-Panx1 (red) antibodies; Panx1 is shown in both neurons (arrowheads) and satellite glial cells (arrows). (**B**) Image obtained from NFH-Cre:Panx1$^{fl/fl}$ trigeminal ganglion immunostained with anti-GS (green) and anti-Panx1 (red) antibodies; in mice with targeted deletion of Panx1 in neurons, Panx1 expression is detected only in satellite glial cells (arrows) and not in neurons (arrowheads). (**C**) Trigeminal ganglion image from GFAP-Cre:Panx1$^{fl/fl}$ mice immunostained with anti-GS (green) and anti-Panx1 (red) antibodies; in mice with targeted deletion of Panx1 from GFAP-positive glial cells, Panx1 is detected in neurons (arrowheads) but not in satellite glial cells (arrows). Images were obtained with a Olympus Fluoview 300 confocal laser scanning microscope equipped with 40× water-immersion lens (0.80 NA), laser lines and appropriate filter sets. Bar: 50 μm. (See Hanstein et al., 2013).

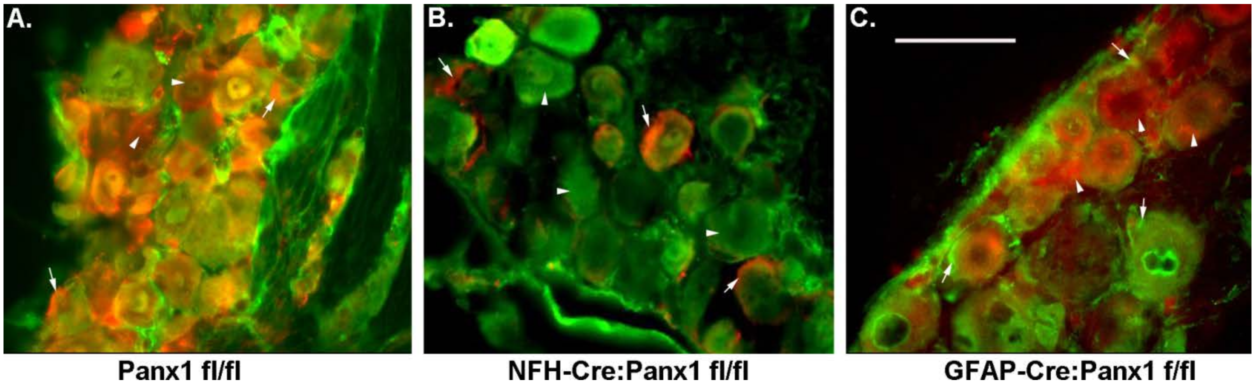

Supplemental Figure 6. Spontaneous ATP release from trigeminal ganglia from control (Panx1f/f) and cell-targeted Panx1 deficient mice at 7 days following SMS injection of saline (Sal) or CFA. Panx1 deletion from GFAP-positive glia or neuron was achieved by breeding Panx1f/f female mice with GFAP-Cre or NFH-Cre males. Values for individual ganglia (two per mouse) from saline or CFA injected mice are shown, with release normalized for saline in each case. Note that ATP release is higher in ganglia from CFA than saline injected controls but not in either GFAP-positive glial or neuronal targeted deletion of Panx1. Mean±SE values are indicated in red, N indicates the number of mice.

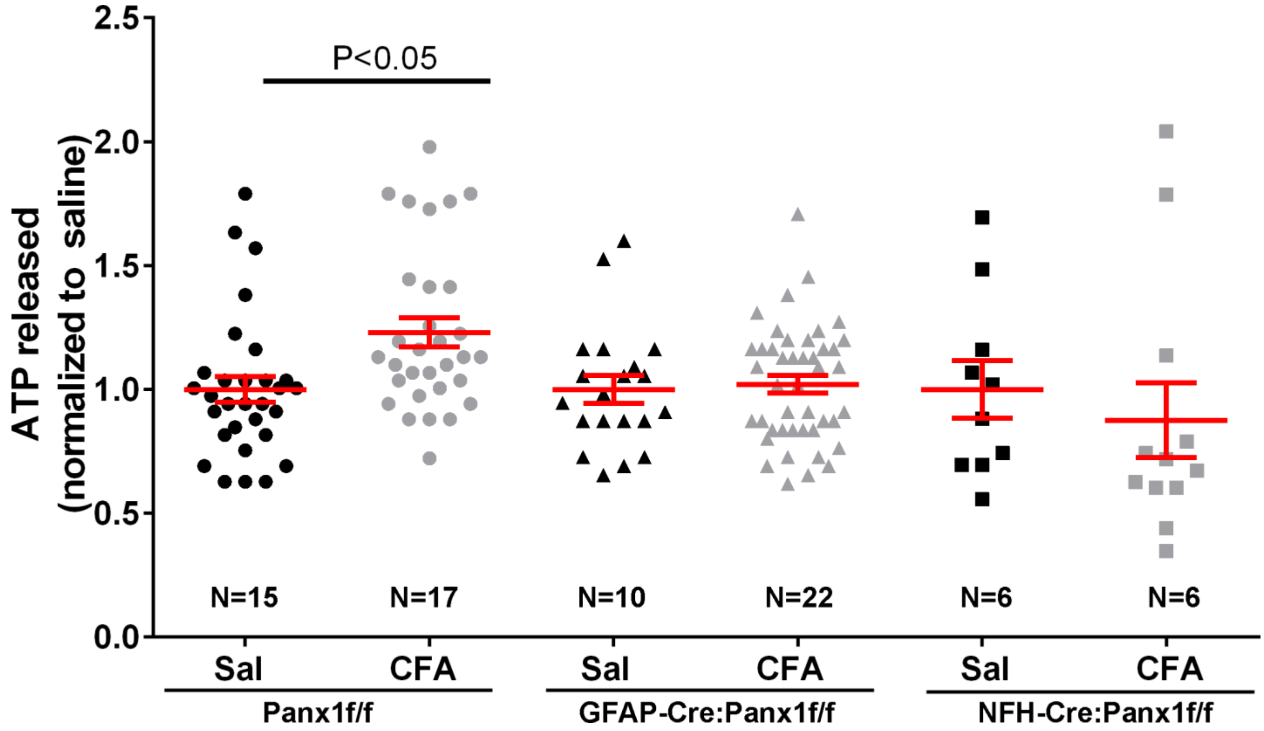

Supplement: Supplementary Information [file srep38266-s1.pdf]
